# A new Landslide Investigation and Simulation Archive through downscaled landslide experiments

**DOI:** 10.1038/s41597-025-05948-z

**Published:** 2025-10-21

**Authors:** Laura Longoni, Alessandro Scaioli, Lorenzo Panzeri, Diego Arosio, Monica Corti, Azadeh Hojat, Monica Papini

**Affiliations:** 1 Dipartimento di Ingegneria Civile, Ambientale e Territoriale, Politecnico di Milano, Milan, 20133 Italy; 2 Dipartimento di Scienze Chimiche e Geologiche, Università degli Studi di Modena e Reggio Emilia, Modena, 41125 Italy; 3 Department of Mining Engineering, Shahid Bahonar University of Kerman, Kerman, 76188 Iran

**Keywords:** Natural hazards, Geophysics, Geology

## Abstract

Downscaled landslide experiments are an effective approach to studying complex interactions between landslide triggering factors. Working at a small scale allows seeing and measuring phenomena that cannot be quantified at a real scale with the same level of accuracy. In addition, small scale tests are useful to analyse the triggering factors separately. In this work, the dataset LISA (*Landslide Investigation and Simulation Archive*), containing more than 50 tests performed over 7 years using the landslide simulator of the Gap^2^Lab in the Lecco Campus of Politecnico di Milano, is presented. This dataset is structured as a database. A database is a powerful tool that allows users to interact completely and comprehensively with the stored data thanks to queries. The data acquired throughout these experiments can be exploited to test and validate landslide models and define rainfall thresholds. The data are available either through the associated database management system (*DBMS*), Microsoft Access, or as a set of flat comma-separated values (*CSV*). The entire dataset is available on the open-access repository Harvard Dataverse.

## Background & Summary

Shallow landslides are highly destructive events. Despite usually involving small areas both in size and depth, once triggered, they can move downslope rapidly, causing fatalities and severe damages to infrastructures in a short time^1^. In the context of risk mitigation, it is crucial to enhance the understanding of shallow landslides by considering all triggering factors and phenomena that influence the dynamic and cinematic behaviour of the slopes.

According to numerous studies^2–4^, the primary triggering factors of shallow landslides, commonly also known as rainfall-induced landslides, are filtration and infiltration^5^. Since the mid-20^th^ century, the frequency and intensity of heavy precipitation events have increased due to climate change, causing in turn hydro-geomorphological events such as floods and landslides^6–9^. Consequently, many authors have analysed water behaviour within landslides using various techniques and approaches^10–14^, including down-scaled experiments^2–4,11,15–18^.

To better understand the process and the interactions between triggering factors and slope parameters, laboratory experiments have been conducted using a landslide simulator, where various soil types, slope angles and rainfall patterns have been used^3^. Thanks to a multidisciplinary approach, involving photogrammetry, geophysical and geological methods^19^, a wide range of devices has been used to monitor water circulation and deformations during the tests to achieve a more accurate comprehension of the slope dynamics. Over 7 years, a large amount of data have been collected from more than 50 experiments performed at the Gap^2^Lab laboratory of Politecnico di Milano. The data have been then organized in a comprehensive dataset to make them easily available. This tool allows users both to quickly obtain key information and to query complete geological, geophysical and photogrammetric datasets.

This dataset marks a significant milestone in the experimental work of our research team, bringing together data and information accumulated over years of investigation. Some of these data have been analysed in previous studies, particularly in the works of Ivanov, V. *et al*.^19,20^. The first one presents a comparative analysis of multiple experiments, while the other focuses on the use of interferometric optical fibres for monitoring shallow landslides. Another notable contribution is by Yordanov, V. *et al*.^21^, which explores the use of photogrammetric techniques to monitor landslide deformation. Finally, the work of Panzeri, L. *et al*.^22^, concentrates on analysing snowmelt processes.

One of the main goals is to provide users with a dataset for the analysis of shallow landslide triggering processes influenced by different rainfall intensities, so that effective considerations regarding triggering thresholds can be done.

Moreover, the dataset includes tests with additional features for analysing seasonal phenomena, such as snowmelt, and factors linked to climate change that may increase the likelihood of landsliding. For example, tests using snow cover and a buried water pipe to simulate snow melting and infiltration have been performed to investigate the effects of processes causing faster snowmelt cycles in mountainous regions^23^. Also, experiments with burned and unburned soils collected from a conifer forest have been performed to quantify the direct influence of wildfires on hydrology and slope stability^24,25^, independent of the vegetation coverage.

## Methods

### The landslide simulator

**Fig. 1:**
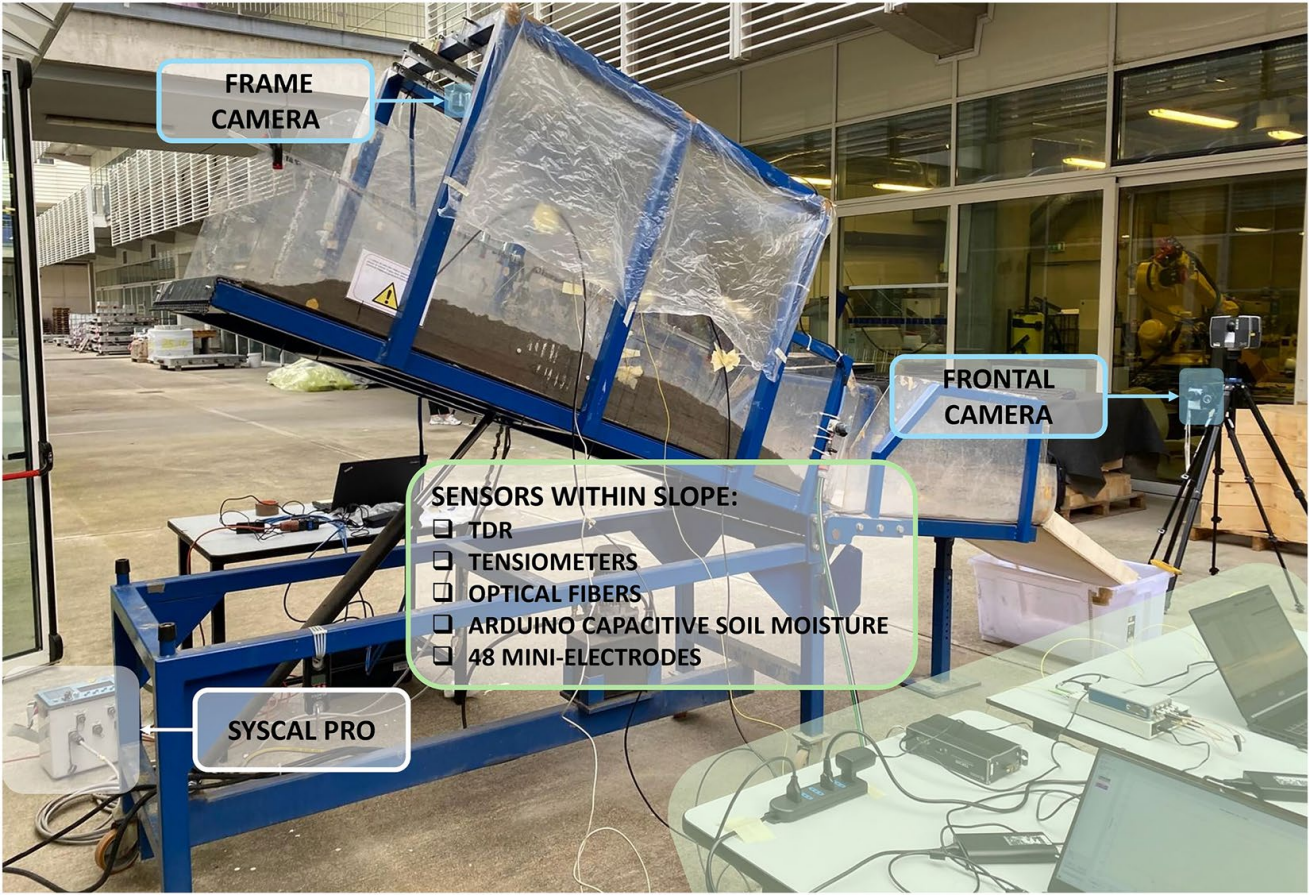
The usual setting of the experiments: the landslide simulator and the monitoring instruments.

Downscaled laboratory tests have been performed on a simulator to study the different phases of slope failure in a controlled environment, analysing the triggering factors and the parameters describing the phenomenon. Various instruments were used to monitor processes involving soil such as water infiltration and ground deformation (Fig. 1).

**Fig. 2:**
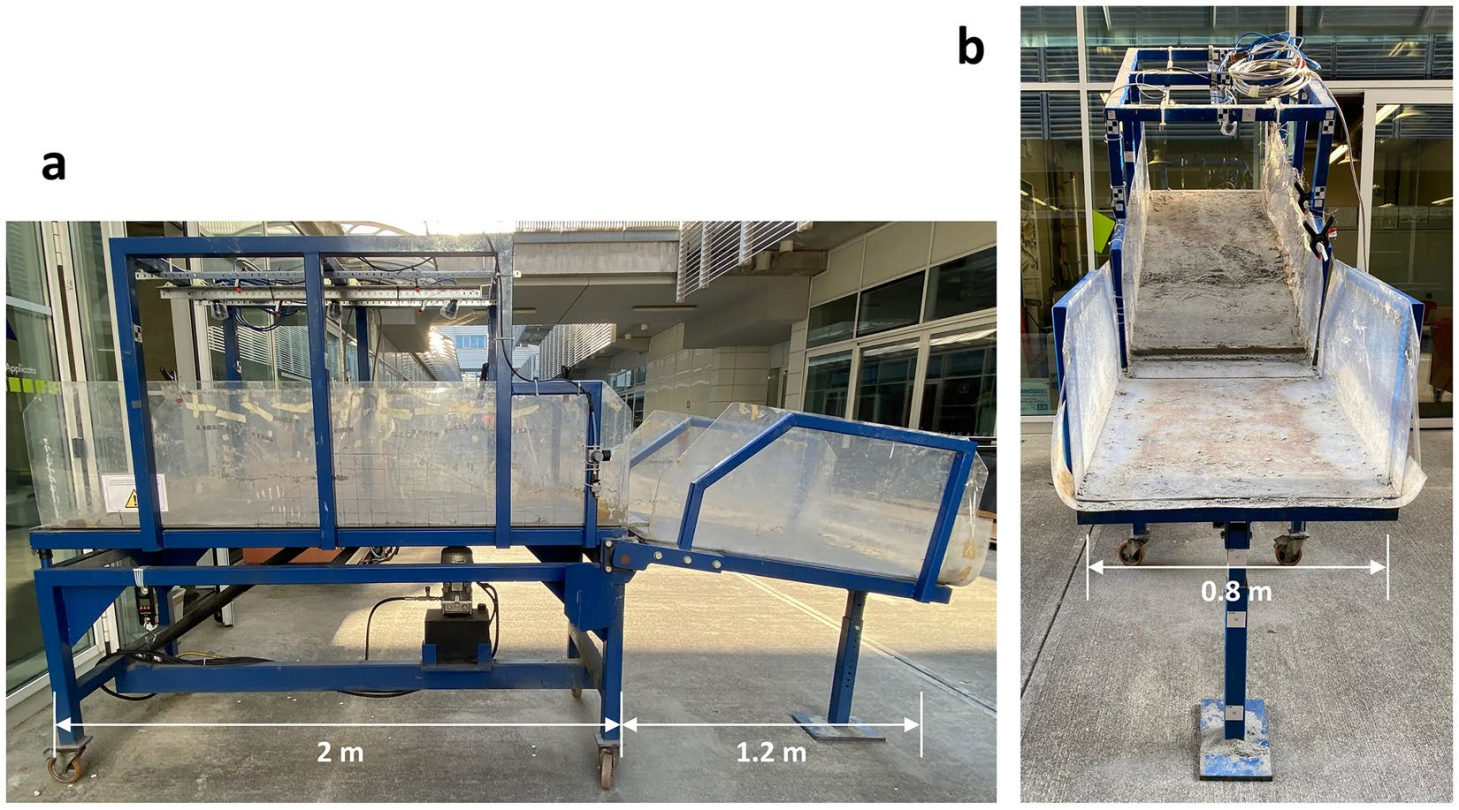
(**a**) Side view of the landslide simulator in the Gap^2^Lab in Lecco Campus of Politecnico di Milano. (**b**) Front view.

The simulator is available in the Gap^2^Lab (*Laboratorio di Geologia e Geofisica Applicata*) at the Lecco Campus of Politecnico di Milano. This tool was designed to reproduce downscaled landslide mechanisms for testing purposes^3,19,26^. The simulator features a flume composed of two rectangular steel surfaces, one fixed and the other tiltable with size 0.8 × 2 m, and plexiglass side walls (Fig. 2a). The soil is placed on the tiltable surface while keeping it horizontal^12^ (Fig. 2b). Then, the slope angle (α) is set using a motorized system up to a maximum value as high as 45° (Table 1).

**Table 1:** Values of the parameters considered in the landslide simulation tests.

	MIN VALUE	MAX VALUE	AVERAGE	MEDIAN	STANDARD DEVIATION
**P [mm/h]**	12	122	60.95	54.74	24.95
**VWC**_**i**_ **[−]**	0.04	0.37	0.14	0.12	0.08
**α [°]**	25	40	34.26	35	5.19
**Longer base [m]**	1.4	2	1.9	1.94	0.28
**Shorter base [m]**	1.2	1.9	1.52	1.5	0.25
**Height [m]**	0.11	0.18	0.15	0.15	0.02
**Weight [kg]**	197.7	322.29	273.39	290	51.82

At first, the soil to be used is placed into buckets, the initial volumetric water content (VWC_i_) is measured with a Time Domain Reflectometry (TDR) probe and, if necessary, adjusted (Table 1). Before placing the soil on the landslide simulator, each bucket is weighed, so that the total weight of the involved material is known.

**Fig. 3:**
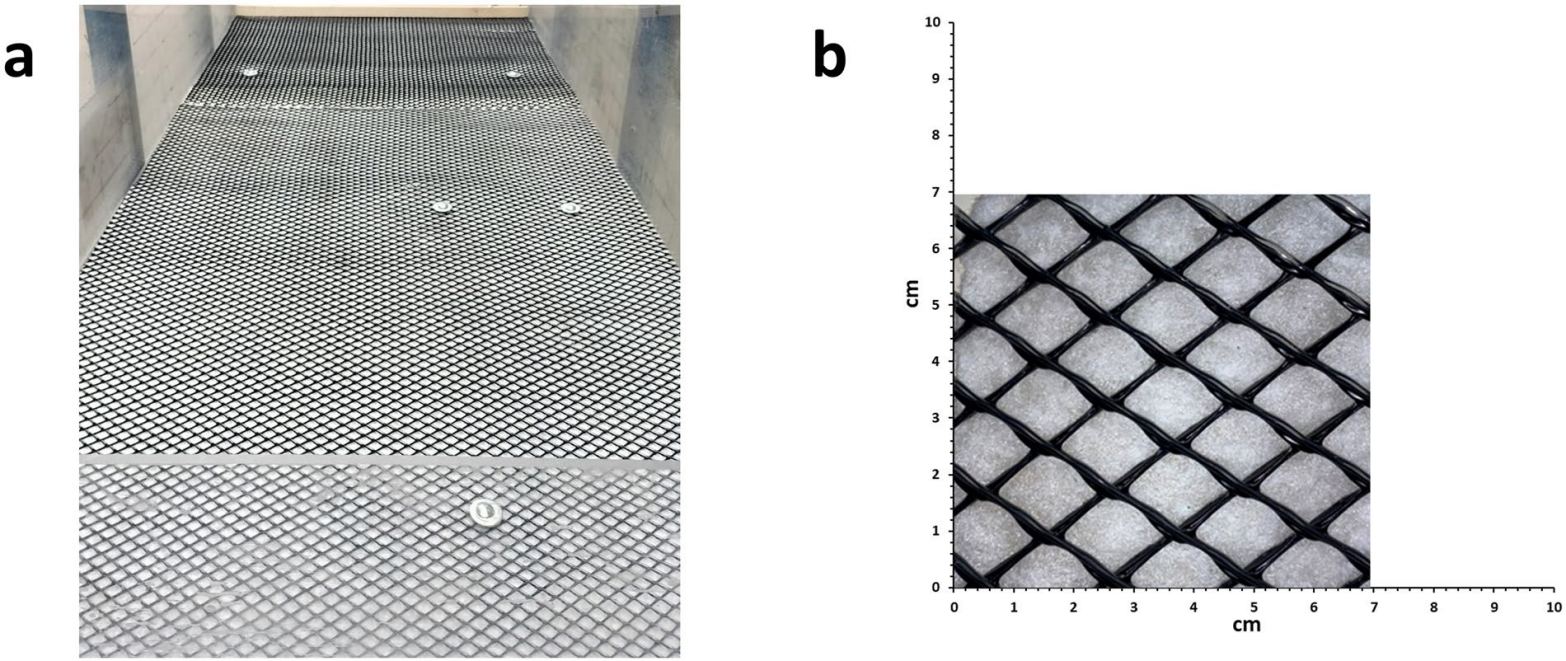
(**a**) The geogrid High Friction Tenax HF installed on the landslide simulator. (**b**) Close up of the geogrid.

On tiltable surface, a geogrid (High Friction Tenax HF by TENAX) was installed to ensure basal friction, preventing unwanted soil slippage. This geogrid (Fig. 3) is made of high-density polyethylene/ethylene vinyl acetate (HDPE/EVA) polymers, whose friction angle is up to 36°^27^.

**Fig. 4:**
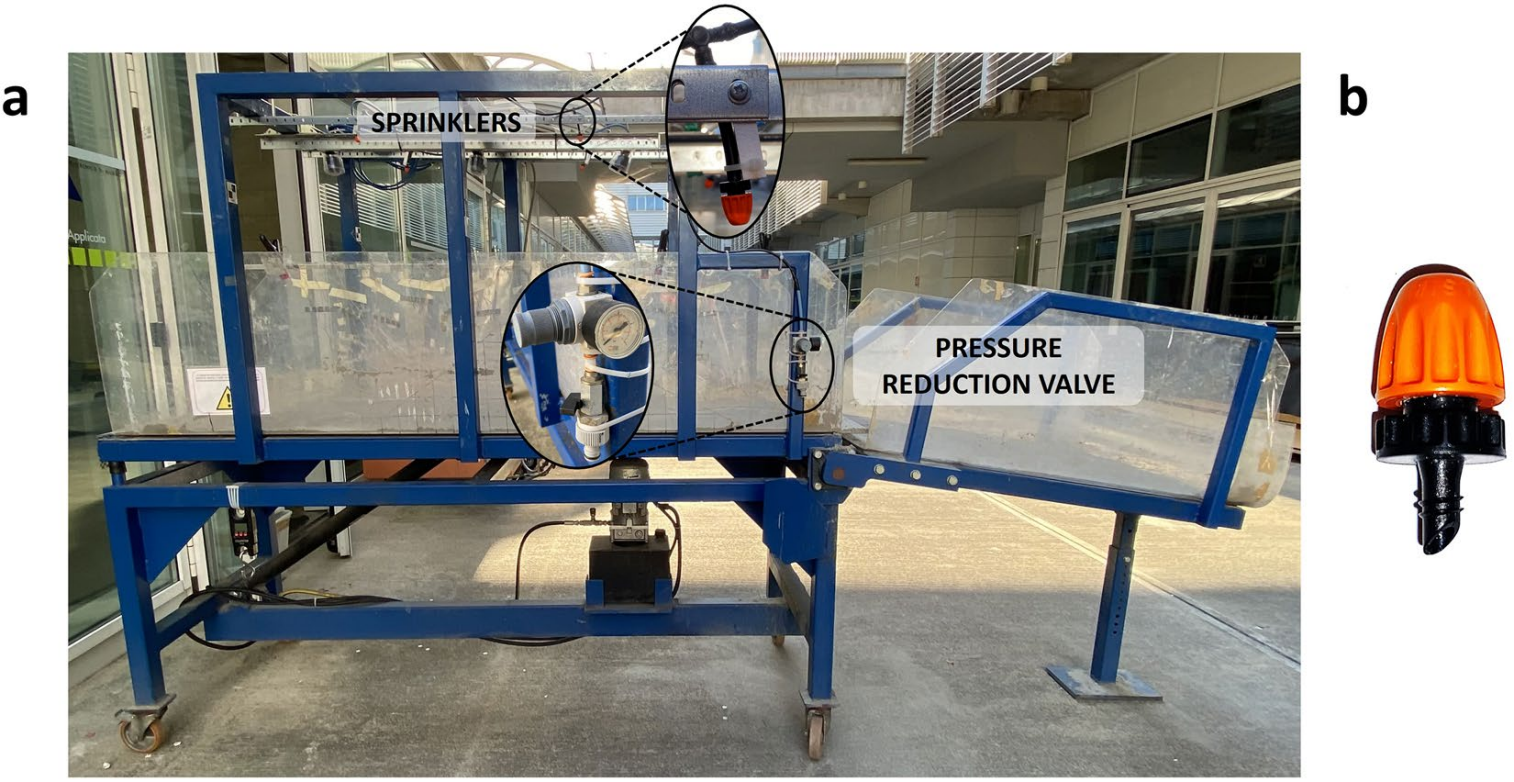
(**a**) Rainfall simulation equipment installed on the landslide simulator with close-ups of a sprinkler and the pressure reduction valve. (**b**) Detail of a sprinkler.

**Fig. 5:**
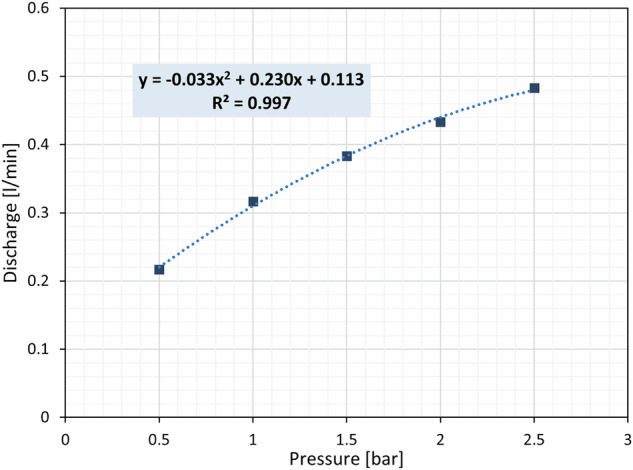
Sprinkler pressure-discharge curve obtained by interpolating values provided by the manufacturer.

A set of sprinklers is used to simulate rainfalls with different intensities, up to 20.6 mm/h per nozzle (Fig. 4a). The sprinklers are installed along two parallel rows on the top structure of the simulator, 0.6 m above the tiltable surface to supply a water spray as much homogenous as possible. The current model of irrigation nozzles, by Claber (Fig. 4b), was chosen to avoid any erosive action by the water droplets. The sprinklers’ water discharge is controlled by a pressure reduction valve, while the resulting rainfall intensity (P) can be estimated through the pressure-discharge characteristic curve provided by the manufacturer (Fig. 5).

Rainfall patterns vary among the experiments. Rainfall can be continuous throughout the test, or one or more pauses can be present (Fig. 7). In the tests, the minimum P of 12 mm/h is used to simulate common rainfall events, while the maximum value of 122 mm/h simulates extreme rainfall events (Table 1).

**Fig. 6:**
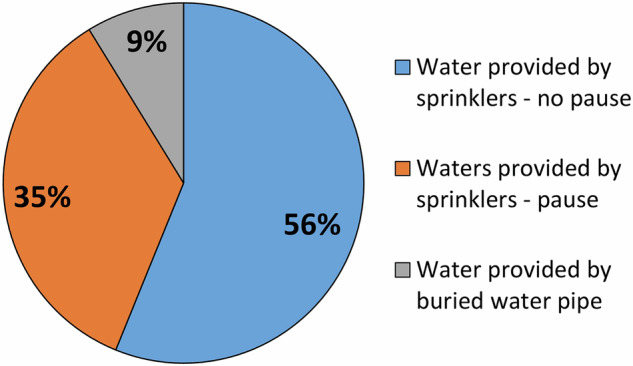
Pie chart showing percentages of experiments with water provided by sprinklers (with and without a pause) and by a buried water pipe.

For some tests, a buried water pipe was used to increase the groundwater level within the slope (Fig. 6).

**Fig. 7:**
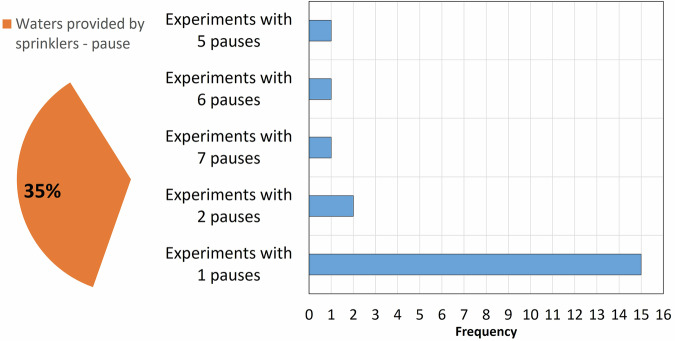
Bar chart listing the tests with water provided by sprinkles according to the number of pauses.

Generally, two different triggering factors can be simulated: rainfall or groundwater flow, which can be generated by sources such as snowmelt^22,23^ or, in rare cases, the failure of water pipes.

The cross-section of the landslide body can be usually approximated to a trapezoidal prism. It is constructed with at least a 10:1 length/depth ratio to fulfil the infinite slope assumptions and minimize the effect of lateral boundaries (Table 1).

### Characteristics of the soils

Different soil materials, including sand, clay, gravel, and sandy loam, were used in the experiments. All the used soils are commercial materials, except for sandy loam samples that were collected from two coniferous forest in northern Italy, where wildfires occurred in January 2019^25^ and February 2022. Samples were taken from both burned and unburned areas, hereafter referred to as burned soil and natural soil, respectively, to assess the influence of wildfires on the slope hydrology. In the experiments, these soils were used to build only the surface layer of the landslide body.

Table 2 contains the value ranges of the most relevant properties describing the considered soil. It includes the porosity n, the void index e, and the hydraulic conductivity k_s_.

**Table 2:** Most relevant properties of the considered soils.

SOIL	n min [−]	n max [−]	e min [−]	e max [−]	ks min [m/s]	ks max [m/s]
Sand	0.44	0.62	0.786	1.632	3.15E-04	4.02E-02
Clay	0.56	0.56	1.273	1.273	1.00E-09	1.00E-09
Burned soil	0.49	0.89	0.961	8.091	7.52E-04	7.52E-04
Natural soil	0.48	0.55	0.923	1.222	5.29E-06	5.29E-06

**Fig. 8:**
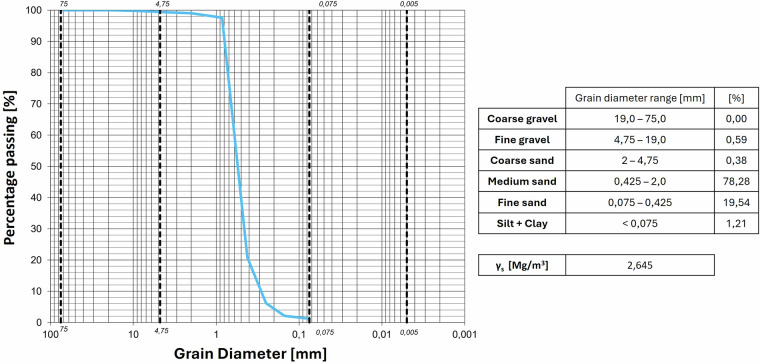
Granulometric curve of the Sand.

**Fig. 9:**
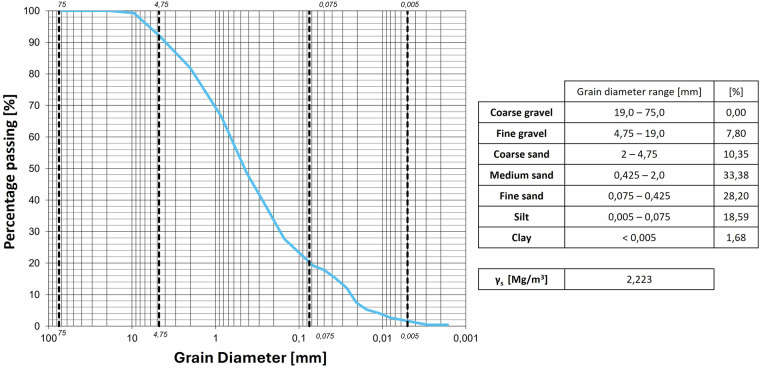
Granulometric curves of the Site 1 Burned soil.

**Fig. 10:**
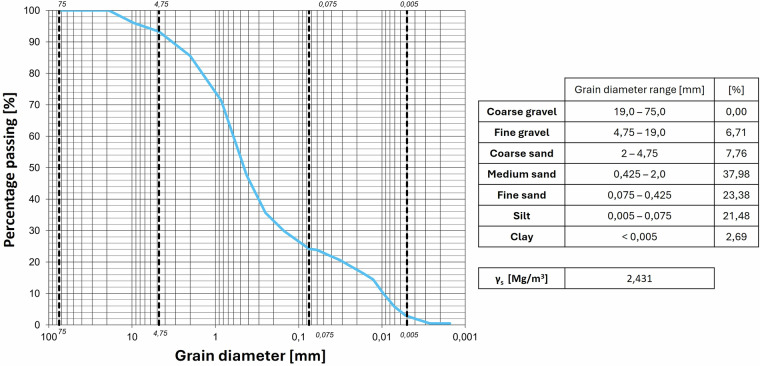
Granulometric curves of the Site 2 Burned soil.

**Fig. 11:**
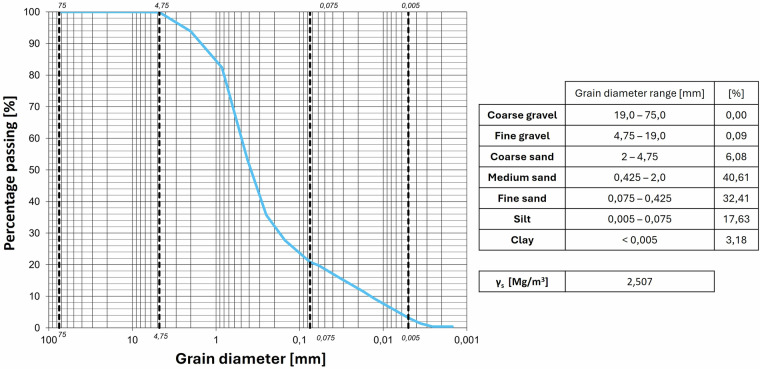
Granulometric curves of Natural soil (site 1).

The granulometric curves of the soils used are reported in Figs. 8–11. Table 3 lists the granulometry parameters D_10_, D_50_, D_60_.

**Table 3:** Granulometry parameters of materials used in the tests.

SOIL	D_10_ [mm]	D_50_ [mm]	D_60_ [mm]
Sand	0.3	0.54	0.6
Burned soil (site 1)	0.023	0.43	0.62
Burned soil (site 2)	0.0092	0.43	0.6
Natural soil (site 1)	0.013	0.4	0.5

The clay used belongs to the LECA type (Lightweight Expanded Clay Aggregate), industrially manufactured by Laterlite S.p.A. For detailed technical specifications, please refer to the Laterlite website^28^.

**Fig. 12:**
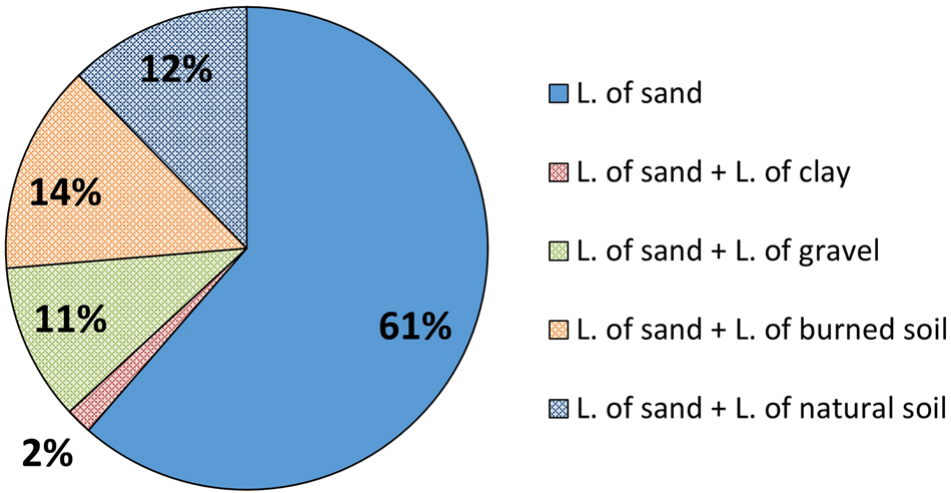
Pie chart showing percentages of experiments according to the involved material. The percentages with hatched fill refer to experiments in which the landslide body was constructed by superimposing homogeneous layers of different materials.

For about 40% experiments, the landslide body was prepared by overlapping homogeneous layers of the different available materials (Fig. 12). As a result, the effect of layers with different grain sizes was considered.

### Monitoring system

A set of instruments was used to monitor the processes during the experiments: TDR for volumetric water content (VWC), tensiometers for temperature and pore water pressure, Arduino probes for soil moisture, optical fibres for slope deformation, optical cameras for surface deformations, Electrical Resistivity Tomography (ERT) for electrical resistivity. Also, superficial runoff was collected and measured over time in several experiments to verify water balance and to quantify the infiltration.

**Fig. 13:**
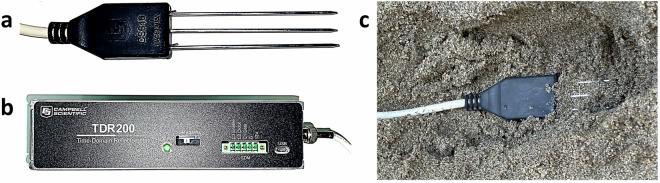
(**a**) Detail of the TDR probe. (**b**) The TDR acquisition system. (**c**) The TDR probe installed within the landslide body.

The TDR200 system produced by Campbell Scientific (Fig. 13) measures the variation of electromagnetic impedance along the transmission line and estimates the VWC that is related to the difference in dielectric permittivity between solid, air, and liquid phases^29–31^. In particular, the used TDR probe (CS640) estimates the average VWC content within a 15 cm-diameter circle centred on the probe. In the dataset, the TDR probe is identified as *TDR_downslope*, *TDR_center_slope* or *TDR_upslope*, based on its location within the landslide body during the test.

**Fig. 14:**
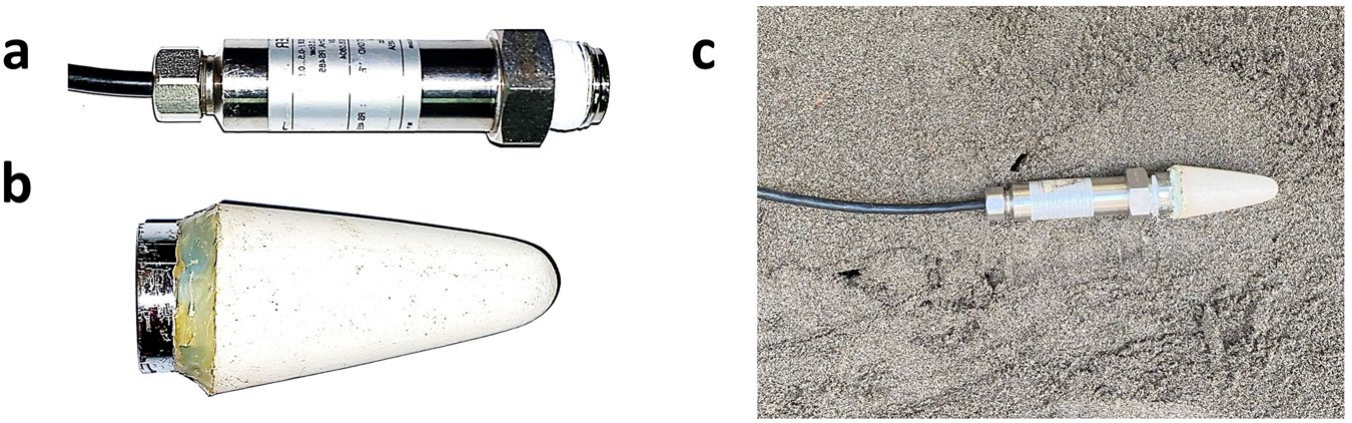
(**a**) The tensiometer without the porous head. (**b**) Detail of the porous head. (**c**) The tensiometer installed within the slope.

The used tensiometers belong to Series 35 X produced by Keller (Fig. 14a) and were customized to measure pore water pressure thanks to a ceramic porous head, produced by TIMESETL, that was specifically added for this purpose (Fig. 14b).They measure the temperature in degrees Celsius (*°C*) and the pore water pressure in *kPa*. The piezoresistive sensor measures positive or negative pressure values based on soil saturation conditions: negative values (suction) indicate partial saturation, while positive values indicate saturated soil conditions.

A maximum of three probes have been installed within the landslide body. However, to avoid excessive disturbance of the slope, only two tensiometers were usually installed.

**Fig. 15:**
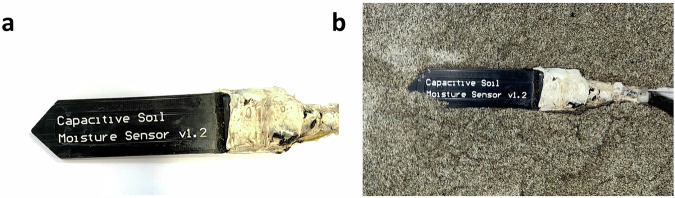
(**a**) The ARDUINO capacitive soil probe. (**b**) The probe installed within the slope.

ARDUINO capacitive soil moisture probes (v1.2) (Fig. 15) are low-cost sensors that provide data about the capacitive properties of the soil during the experiment, which can be related to soil moisture. As for tensiometer, although six ARDUINO probes were available, generally only five were placed to minimize soil disturbance.

**Fig. 16:**
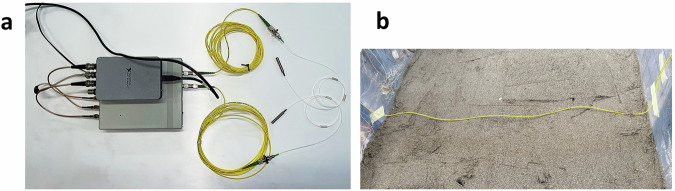
(**a**) Interferometric optical fibre sensors with the interferometer and the power supply. (**b**) The optical fibre installed within the slope.

The deployed interferometric optical fibre sensors (Fig. 16) are produced by Cohaerentia, a startup of Politecnico di Milano. These optical fibres are continuous and coherent, meaning that their working principle relies on the coherence of the light source: the light waves maintain a fixed phase relationship. This coherence is crucial for detecting small phase shifts caused by strain or temperature changes. Any perturbation in the fibre affects the phase of the light signal travelling through the fibre. These changes are reflected and compared to the original signal in terms of angular difference measured in *rad*. In most tests using optical fibre technology, only one fibre was placed inside the landslide body. More fibre sensors were installed only during experiments specifically aimed at testing optical fibre performance.

The position of each probe within the landslide body is shown in the instrument location sketches included in the dataset. These sketches are displayed when the database automatically generates a report for a specific experiment. The instruments were placed to provide a comprehensive and detailed overview of the internal processes occurring within the simulated landslide. Specifically, when homogeneous layers of different materials were used, the probes were placed to capture the dynamics both within each layer and at their interfaces.

**Fig. 17:**
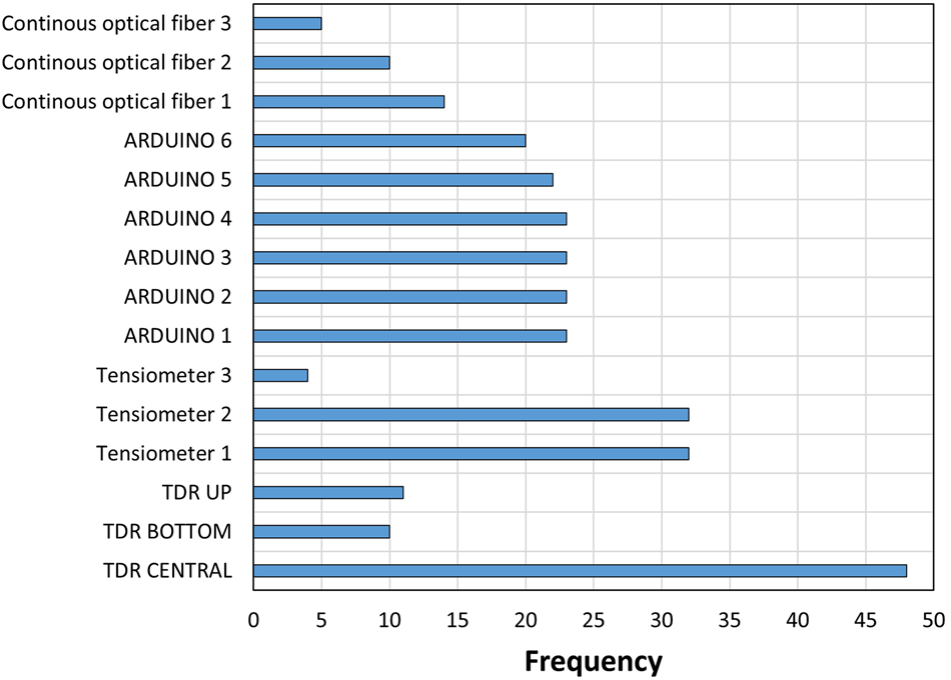
Occurrence of the deployment of the sensors across all experiments.

Figure 17 shows the occurrence of the deployment of the different sensors across all experiments.

The ERT measurements were performed using the IRIS Syscal Switch Pro georesistivimeter, available in the geophysical laboratory of the Politecnico di Milano, with 48 electrodes attached to two multipolar cables. To perform the ERT measurements at the laboratory scale, two small-scale multipolar cables were prepared with the maximum distance of 5 cm between the individual cable take-outs. Each wire of the multipolar cable was attached to a mini stainless-steel electrode (2 cm long with 2 mm diameter) at one end and to a Syscal Switch Pro compatible connector at the other end.

**Fig. 18:**
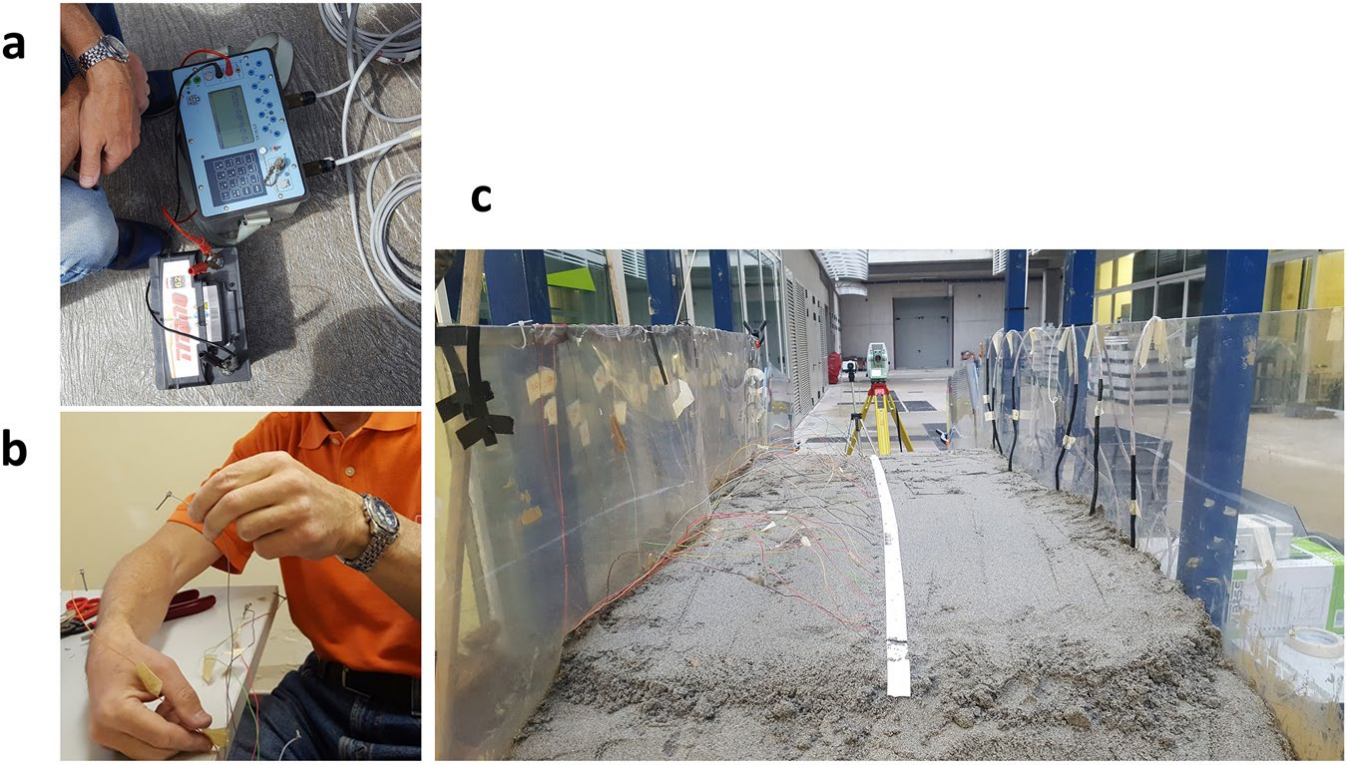
(**a**) The IRIS Syscal Switch Pro georesistivimeter. (**b**) Mini electrodes being attached to lab multipolar cables. (**c**) 48 electrodes inserted in the soil along the ERT line before being covered with a top shallow layer.

The ERT monitoring was integrated into landslide simulation tests because ERT method has proved to be an efficient element to be included in hydrogeological risk mitigation strategies. The main advantage of the ERT method is its potential to monitor changes in water content of the material^13,32,33^. ERT measurements in our landslide simulation experiments were mostly performed using the Wenner electrode configuration. A few experiments were also performed using the Wenner-Schlumberger array. The cable was deployed along the main axis in the middle of the landslide body, mostly using an electrode spacing of 3 cm (Fig. 18). Once deployed, the electrodes were covered with about one or two centimeters of soil, simulating a real long-term installation where the electrodes are usually buried in a trench. To reduce the time gap between different ERT surveys, a major part of data acquisitions was carried out using the High-speed option of the georesistivimeter. Using this option, the measurements at each point (i.e., for each single quadrupole) are not repeated and the acquisition time for the Wenner array using 48 electrodes and 12–15 depth levels was reduced to about 3.5–4 min.

**Fig. 19:**
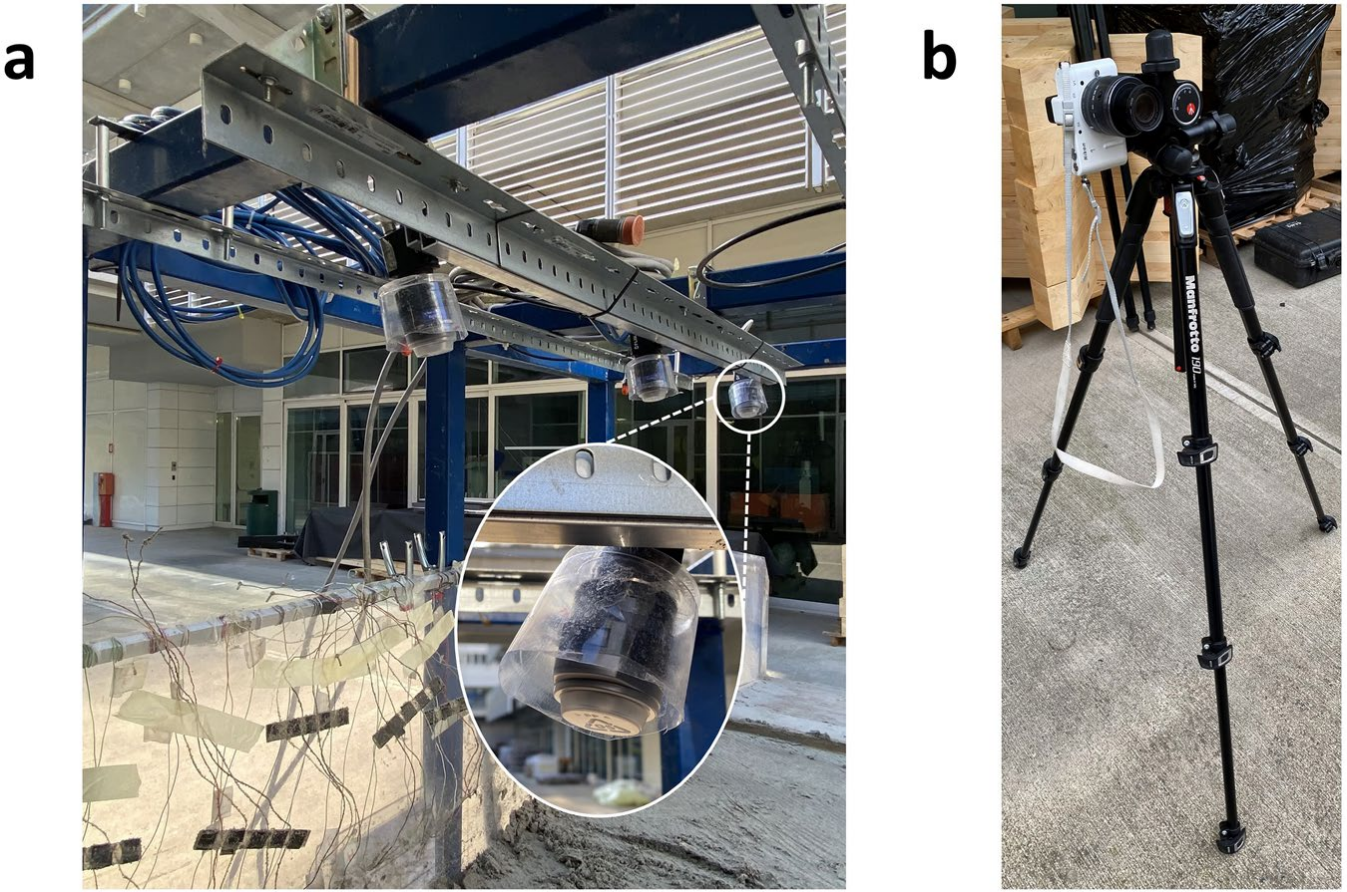
(**a**) The three cameras installed on the top frame of the landslide simulator. (**b**) The camera in front of the simulator.

Different photogrammetric settings were employed across the experiments (Fig. 19). Cameras were installed directly on the landslide simulator (Fig. 19a) and/or in front of it, to better appreciate all the stages of the slope failures^34^ (Fig. 19b).

## Data Records

### Detailed overview of LISA dataset

**Fig. 20:**
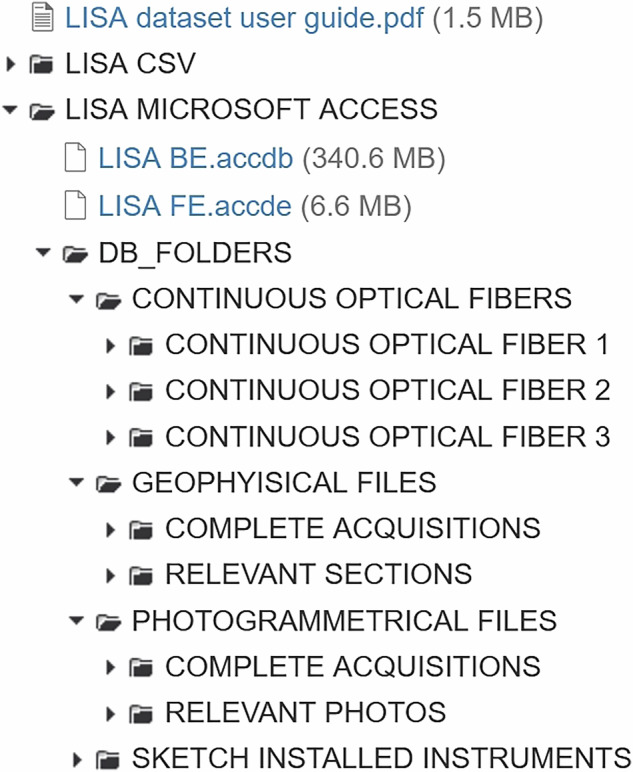
Data Organization Structure in the Harvard Dataverse.

LISA is a dataset containing the data related to 57 experiments. It can be fully and freely downloaded from the open-access repository Harvard Dataverse^35^ via https://dataverse.harvard.edu/dataset.xhtml?persistentId=doi:10.7910/DVN/0H4AVF with license CC BY. Figure 20 shows how the data and relevant information have been organized in the Harvard Dataverse repository.

The data are available in two formats: Microsoft Access files or a set of CSV files.

The first option is to use the LISA dataset as a database, paired with a DBMS (Microsoft Access). This allows many built-in functionalities, mainly queries and reports, which simplify the database’s use.

In the folder *LISA MICROSOFT ACCESS*, the Microsoft Access files ***LISA BE*** and ***LISA FE*** are available, together with the *DB_FOLDERS* folder. This folder contains the following subfolders: *PHOTOGRAMMETRICAL FILES*, *SKETCH INSTALLED INSTRUMENTS*, *GEOPHYSICAL FILES*, *CONTINUOUS OPTICAL FIBRES*.

***LISA BE*** is the back-end (BE) designed to store all the information tables, and ***LISA FE*** is the front-end (FE), i.e. the user interface. It contains all the preset queries and reports that can be easily exploited to perform data analysis.

The *LISA CSV* folder contains the database tables in the CSV format. These should be used when working with a DBMS other than Microsoft Access.

A User guide and additional information about the data and its usage are provided in the repository.

### Data example

**Fig. 21:**
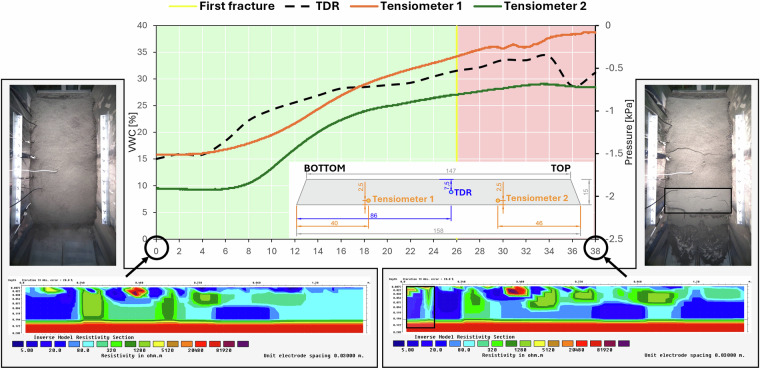
VWC and pressure measured by the two tensiometers. The photos and the ERT sections refer to the beginning of the experiment (left) and to the post-collapse phase (right). A sketch (in cm) showing the positions of the TDR probes and tensiometers within the landslide body is also provided; TOP refers to the upper part of the landslide body when the tiltable plane is inclined, and BOTTOM refers to the lower part.

This paragraph presents an example of the measurements recorded by some probes and ERT acquisitions during a specific experiment (Fig. 21). In particular, the experiment considered is labelled ID 4 and its details are listed in Table 4.

**Table 4:** Experiment ID 4 details.

**General information**	Starting time: 15:41
	Ending time: 16:19
**Slope inclination**	35°
**Rainfall simulation**	Rainfall provided by 4 sprinklers (P = 57.5 mm/h)
**Time first fracture**	26 min
**Soil properties**	Sand:
	- n = 0.48
	- ks = 4.02e^−4^ m/s
	- VWC_i_ = 15%

Examples of more in-depth analysis of landslide simulator experimental data can be found in past articles^19–23^.

## Technical Validation

The general experimental procedure described in the previous paragraphs was followed rigorously throughout the tests to ensure standardization. However, each experiment presents unique characteristics related to different testing conditions (i.e., rainfall/water supply and slope).

### Agreement between measurements

The data acquired by instruments measuring quantities that are dimensionally different but are related to the same process, like water circulation, were compared to verify the expected agreement between measurements.

Table 5, for example, shows the comparison between TDR, tensiometer and ERT measurements for experiment ID 4. The setup of this experiment is one of the simplest, with the slope composed of sand only and water provided by continuous rainfall.

**Table 5:** ERT sections and sensor measurements related to experiment ID 4. t_REL_ is the time when rainfall starts.

t_REL_ [min]	ERT [Ω∙m]	TDR VWC [-]	Tensiometer 1 Pore pressure [kPa]	Tensiometer 2 Pore pressure [kPa]
**0**	Tensiometer 1 (downslope): [10-20], TDR (center): [40-80], Tensiometer 2 (upslope): [80-160]	0.15	−1.51	−1.91
**28**	Tensiometer 1 (downslope): [5-10], TDR (center): [20-40], Tensiometer 2 (upslope): [40-80]	0.32	−0.29	−0.77

As observed in the ERT section at the TDR location, resistivity decreases 28 min after the start of the test. This corresponds to an increase in VWC measured by the TDR. However, the material surrounding the TDR probe does not seem to be fully saturated because, quantitatively, the TDR measures a VWC value lower than the porosity and, qualitatively, the ERT section shows higher saturation areas (dark blue colours) with lower resistivity.

The data recorded by tensiometers align well with ERT surveys. Both probes measure an increase in pore pressure after 28 minutes, while the ERT surveys confirm this trend through a resistivity decrease in those regions. The relative difference between tensiometer 1 and 2 is confirmed by ERT surveys. Geoelectrical acquisitions reveal that the area near tensiometer 1 is constantly characterized by a higher degree of saturation compared to the area near tensiometer 2.

### Agreement between experiments

A complete validation of the laboratory tests performed on the landslide simulator requires thorough checks on the repeatability of the tests, the consistency of the results and the comparisons with theoretical models or observed real-world data. In the absence of suitable real cases for validation, the technical validation provided in this section presents a comparison of some representative experiments to show the consistency among them. There is also an analysis of the differences between experimental observations and the results from an analytical approach.

Tests under identical conditions were not performed, and therefore it is not possible to strictly evaluate the repeatability of the experiment.

At the present stage, LISA does not include tests that can be entirely overlapped. We have not performed tests that can be considered identical in every aspect because one of the major aims of our experimental campaigns has been to investigate the influence of a single parameter on the outcome of the tests.

For consistency analyses, 12 experiments with slope composed of sand only and with continuous rainfall were selected.

Table 6 confirms that when experiments are performed with the same VWC_i_ and P, the time to first crack formation (t_fc_) is inversely proportional to the α. Conversely, if VWC_i_ and α are fixed, the first crack occurs earlier when P is higher, as shown in Table 7. Finally, when P and α are constant, an increase of VWC_i_ leads to a shorter t_fc_ (Table 8).

**Table 6:** Experiments with the same VWC_i_ and P, but different α. See text for details.

Fixed variables:	VWC_i_, P			
ID	VWC_i_ [−]	P [mm/h]	α [°]	t_fc_ (measured) [min]
5	0.15	57.5	**33**	**27**
4	0.15	57.5	**35**	**25**

**Table 7:** Experiments with the same VWC_i_ and α, but different P. See text for details.

Fixed variables:	VWC_i_, α			
ID	VWC_i_ [-]	P [mm/h]	α [°]	t_fc_ (measured) [min]
4	0.15	**57.5**	35	**26**
27	0.15	**66.01**	35	**14**

**Table 8:** Experiments with the same P and α, but different VWC_i_. See text for details.

Fixed variables:	P, α			
ID	VWC_i_ [-]	P [mm/h]	α [°]	t_fc_ (measured) [min]
***Configuration of P and α n1***				
13	**0.073**	72	25	**31**
12	**0.13**	72	25	**23**
***Configuration of P and α n2***				
51	**0.109**	88	40	**19**
50	**0.226**	88	40	**15**

Another approach that can be used to validate the experiments is to compare the results with numerical/analytical methods and/or theoretical models. A comparison between the experimental setting and the results gained after the application of the limit equilibrium model proposed by Montrasio and Valentino^36,37^ (*Shallow Landslides Instability Prediction – SLIP*) is reported.

Even if the SLIP model approach may be oversimplified to fully capture the complexity of the considered experiments, it remains a valuable tool for further validating the landslide simulations. Table 9 shows that there is generally a good agreement between the model outputs and the observed test results.

**Table 9:** Comparison in terms of absolute difference (δ_abs_) between SLIP model and experimental observations.

ID	t_fc_ SLIP MODEL	t_fc_ MEASURED	δ_abs_ [min]
4	30	26	4
5	31	27	4
4	30	26	4
27	25	14	11
12	25	23	2
13	30	31	1
50	22	15	7
51	22	19	3
		**μ**	4.7
		**Std Dev**	3.2

### Details about ERT acquisitions

Considering rapid changes in the landslide body during laboratory tests, the high-speed option of the Syscal Switch Pro was used for most data acquisitions to avoid the temporal smear.

This option has the disadvantage of not being able to estimate data errors from the standard deviation of the readings because the same measurements are not repeated. However, the availability of many datasets makes it possible to use the data measured when no changes were expected to set a cutoff value for the resistivity percentage and to differentiate the real anomalies from artefacts caused by noise.

## Usage Notes

This section provides some tips on geoelectrical data processing. After the electrodes and cables are covered, the effect of the layer of the soil above the electrodes needs to be corrected because the equipment firmware calculates the apparent resistivity with the standard geometrical factor (e.g., 2πa for the Wenner configuration). Details on how to correct the raw data for this effect are explained in Hojat A. *et al*.^38^.

Another important issue in processing the ERT data is the anomalous increasing trend observed in all measured resistivity values with depth. The gradual increase in resistivity values with an increase in electrode spacing arise from the effect of the resistive base of the landslide simulator. Therefore, this sharp boundary can be introduced into the inversion algorithm (for example the ‘sharp boundary’ option in RES2DINVx64) to account for sharp changes in resistivity values across this boundary and to obtain accurate resistivity images of the soil layer after inverting the data.

## Data Availability

No custom code was used in the preparation or analysis of the dataset. The ERT data was processed using the RES2DINVx64 software.

## References

[1] Bugnion, L. & Wendeler, C. Shallow landslide full-scale experiments in combination with testing of a flexible barrier. *WIT Transactions on Engineering Sciences* **67**, 161–173 (2010).

[2] Zhang, K., Wang, S., Bao, H. & Zhao, X. Characteristics and influencing factors of rainfall-induced landslide and debris flow hazards in Shaanxi Province, China. *Natural Hazards and Earth System Sciences* **19**, 93–105 (2019).

[3] Ivanov, V. *et al*. Applicability of an interferometric optical fibre sensor for shallow landslide monitoring – Experimental tests. *Eng Geol* **288**, 106128 (2021).

[4] Sitarenios, P., Casini, F., Askarinejad, A. & Springman, S. Hydro-mechanical analysis of a surficial landslide triggered by artificial rainfall: The Ruedlingen field experiment. *Geotechnique* **71**, 96–109 (2021).

[5] Osawa, H., Matsuura, S., Matsushi, Y. & Okamoto, T. Seasonal change in permeability of surface soils on a slow-moving landslide in a heavy snow region. *Eng Geol* **221**, 1–9 (2017).

[6] Kundzewicz, Z. W. *et al*. Le risque d’inondation et les perspectives de changement climatique mondial et régional. *Hydrological Sciences Journal* **59**, 1–28 (2014).

[7] Li, Z. & Fang, H. Impacts of climate change on water erosion: A review. *Earth Sci Rev* **163**, 94–117 (2016).

[8] Crozier, M. J. Deciphering the effect of climate change on landslide activity: A review. *Geomorphology* **124**, 260–267 (2010).

[9] Corti, M., Francioni, M., Abbate, A., Papini, M. & Longoni, L. Analysis and modelling of the september 2022 flooding event in the misa basin. *Italian Journal of Engineering Geology and Environment* **1**, 69–76 (2024).

[10] Moriwaki, H. *et al*. Failure processes in a full-scale landslide experiment using a rainfall simulator. *Landslides* **1**, 277–288 (2004).

[11] Wang, G. & Sassa, K. Pore-pressure generation and movement of rainfall-induced landslides: Effects of grain size and fine-particle content. *Eng Geol* **69**, 109–125 (2003).

[12] Wu, L. Z., Huang, R. Q., Xu, Q., Zhang, L. M. & Li, H. L. Analysis of physical testing of rainfall-induced soil slope failures. *Environ Earth Sci* **73**, 8519–8531 (2015).

[13] Hakro, M. R. & Harahap, I. S. H. Laboratory experiments on rainfall-induced flowslide from pore pressure and moisture content measurements. *Natural Hazards and Earth System Sciences Discussions* **3**, 1575–1613 (2015).

[14] Iverson, R. M., Logan, M., Lahusen, R. G. & Berti, M. The perfect debris flow? Aggregated results from 28 large-scale experiments. *J Geophys Res Earth Surf* **115** (2010).

[15] Huang, C. C. & Yuin, S. C. Experimental investigation of rainfall criteria for shallow slope failures. *Geomorphology* **120**, 326–338 (2010).

[16] Damiano, E. & Olivares, L. The role of infiltration processes in steep slope stability of pyroclastic granular soils: Laboratory and numerical investigation. *Natural Hazards* **52**, 329–350 (2010).

[17] Askarinejad, A., Casini, F., Bischof, P., Beck, A. & Springman, S. M. Rainfall induced instabilities: a field experiment on a silty sand slope in northern Switzerland. *Rivista Italiana di Geotecnica* **3**, 50–71 (2012).

[18] Casini, F., Serri, V. & Springman, S. M. Hydromechanical behaviour of a silty sand from a steep slope triggered by artificial rainfall: From unsaturated to saturated conditions. *Canadian Geotechnical Journal* **50**, 28–40 (2013).

[19] Ivanov, V. *et al*. Investigation on the role of water for the stability of shallow landslides-insights from experimental tests. *Water (Switzerland)* **12** (2020).

[20] Ivanov, V. *et al*. Applicability of an interferometric optical fibre sensor for shallow landslide monitoring – Experimental tests. *Eng Geol* **288** (2021).

[21] Yordanov, V., Truong, X. Q., Corti, M., Longoni, L. & Brovelli, M. A. Application of lucas-kanade dense flow for terrain motion in landslide monitoring application. in *International Archives of the Photogrammetry, Remote Sensing and Spatial Information Sciences - ISPRS Archives***vol. 48** 1089–1096 (International Society for Photogrammetry and Remote Sensing, 2023).

[22] Panzeri, L., Mondani, M., Papini, M. & Longoni, L. Snow melting experimental analysis on a downscaled shallow landslide: a focus on the seepage activity of snow-soil system. *Water (Basel)* **17**, 597 (2025).

[23] Panzeri, L., Mondani, M., Taddia, G., Papini, M. & Longoni, L. Analysis of snowmelt as a triggering factor for shallow landslide. in *International Multidisciplinary Scientific GeoConference Surveying Geology and Mining Ecology Management, SGEM***vol. 22** 77–83 (International Multidisciplinary Scientific Geoconference, 2022).

[24] Abbate, A., Longoni, L., Ivanov, V. I. & Papini, M. Wildfire impacts on slope stability triggering in mountain areas. *Geosciences (Switzerland)* **9**, 417 (2019).

[25] Corti, M., Corti, L., Abbate, A., Papini, M. & Longoni, L. Post-wildfire terrain evolution in an alpine area. in *International Multidisciplinary Scientific GeoConference Surveying Geology and Mining Ecology Management, SGEM***vol. 22** 159–166 (International Multidisciplinary Scientific Geoconference, 2022).

[26] Longoni, L. *et al*. Laboratory tests with interferometric optical fibre sensors to monitor shallow landslides triggered by rainfalls. *Landslides* **19**, 761–772 (2022).

[27] Tenax Geosynthetics. Tenax HF-HF PLUS Technical Data Sheet. https://www.tenax.net/wp-content/uploads/2017/11/Scheda_Tecnica_TENAX_HF-HF-Plus_i.pdf.

[28] Laterlite, S. P. A. Manuale-geotecnica. https://www.leca.it/wp-content/uploads/2020/07/Manuale-geotecnica.pdf (2020).

[29] Topp, G. C., Davis, J. L. & Annan, A. P. Electromagnetic determination of soil water content: Measurements in coaxial transmission lines. *Water Resour Res* **16**, 574–582 (1980).

[30] Topp, G. C., Yanuka, M., Zebchuk, W. D. & Zegelin, S. Determination of electrical conductivity using time domain reflectometry: Soil and water experiments in coaxial lines. *Water Resour Res* **24**, 945–952 (1988).

[31] Bittelli, M. Measuring Soil Water Content: A Review. *Horttechnology* **21**, 293–300 (2011).

[32] Boyd, J. P. *et al*. Practical considerations for using petrophysics and geoelectrical methods on clay rich landslides. *Eng Geol* **334**, 107506 (2024).

[33] Whiteley, J. S., Chambers, J. E., Uhlemann, S., Wilkinson, P. B. & Kendall, J. M. Geophysical Monitoring of Moisture-Induced Landslides: A Review. *Reviews of Geophysics* **57**, 106–145 (2019).

[34] Scaioni, M. *et al*. Some tools to support teaching photogrammetry for slope stability assessment and monitoring. in *International Archives of the Photogrammetry, Remote Sensing and Spatial Information Sciences - ISPRS Archives* vol. 42 453–460 (International Society for Photogrammetry and Remote Sensing, 2018).

[35] Longoni, L. *et al*. LISA. V2. *Harvard Dataverse*10.7910/DVN/0H4AVF (2025).

[36] Montrasio, L. & Valentino, R. Natural Hazards and Earth System Sciences A model for triggering mechanisms of shallow landslides. *Hazards Earth Syst. Sci* **8**, 1149–1159 (2008).

[37] Montrasio, L. & Valentino, R. Modelling Rainfall-induced Shallow Landslides at Different Scales Using SLIP - Part i. in *Procedia Engineering***vol. 158** 476–481 (Elsevier Ltd, 2016).

[38] Hojat, A., Tresoldi, G. & Zanzi, L. Correcting the Effect of the Soil Covering Buried Electrodes for Permanent Electrical Resistivity Tomography Monitoring Systems. in *4th Asia Pacific Meeting on Near Surface Geoscience & Engineering* 1–5. 10.3997/2214-4609.202177070 (European Association of Geoscientists & Engineers, 2021).

